# Activating Mechanisms Through Employee-Driven Innovation: Comment on "Employee-Driven Innovation in Health Organizations: Insights From a Scoping Review"

**DOI:** 10.34172/ijhpm.8612

**Published:** 2024-09-16

**Authors:** Carolyn Steele Gray

**Affiliations:** ^1^Science of Care Institute & Lunenfeld-Tanenbaum Research Institute, Sinai Health, Toronto, ON, Canada.; ^2^Institute of Health Policy, Management and Evaluation, Dalla Lana School of Public Health, University of Toronto, Toronto, ON, Canada.; ^3^Canada Research Chairs Program, Toronto, ON, Canada.

**Keywords:** Innovation, Healthcare Workforce, System Transformation, Implementation, Change Mechanisms, Organizational Behaviour

## Abstract

Caddedu and colleagues’ paper "Employee-Driven Innovation in Health Organizations: Insights From a Scoping Review," presents findings regarding the state of the literature around employee-driven innovation (EDI). In uncovering the who, what, and how of EDI in healthcare organizations the authors suggest that embracing EDI at an organizational level may be a key to supporting larger system transformation efforts. This commentary builds on this contention suggesting that to help realize that broader vision, attention should be paid to the overlapping implementation mechanisms around empowerment, adaptability, learning, and meaning and value that drive both processes. Finally, it is suggested that what may be most powerful about EDI is its ability to bring joy and vitality back to a healthcare workforce that is currently in crisis.

 The recently published paper, “Employee-Driven Innovation in Health Organizations: Insights From a Scoping Review,” attends to this important and growing activity within healthcare organizations.^1^ The scoping review seeks to better understand employee-driven innovation (termed EDI by the authors) through an exploration of four questions: (1) What is EDI in health organizations and which frontline actors are involved?; (2) What are the characteristics of the EDI process?; (3) What contextual factors enable or impede EDI?; and (4) What benefits does EDI bring to health organizations? The review offers definitional clarity on the subject, defining it was either a participatory process, a learning process or an innovation outcome, all of which point the importance of empowering frontline workers to have more control over the activities, processes and outcomes of innovation in their organization.

 In responding to the last three questions of the review the authors generate a very useful diagram which outlines the processes, enablers, and benefits of EDI. Important in these findings is where innovation is generated (from either top-down, bottom-up or via hybrid of these two), the micro, meso, and macro level contextual enablers to EDI processes, and the benefits that can be realized at individual, organizational, and system levels. The authors note an important knowledge gap around macro-level factors, however miss an opportunity to engage in the space between organizations and macro environment, as interorganizational learning and isomorphic pressures may play a role in EDI adoption.^2^

 An inspection of these factors that are identified in this review brings to mind learning from realist theory and implementation science around the notion of mechanisms that drive change. Caddedu et al suggest that embracing EDI may be a method through which larger system transformation may occur, which could be well realized by noting the important overlaps between the mechanisms that underpin the framework presented in their paper, and the mechanisms that have been found to drive large-system transformation.^3,4^ This commentary draws on the implementation science and realist literatures to suggest how the transformative power of EDI could be realized by activating change mechanisms.

## Understanding Mechanisms

 Drawing from realist and implementation science literatures, mechanisms can be defined as “processes or events through which implementation strategies operate to affect one or more implementation outcomes; or how or why strategies work”^5^ (p. 2). Importantly, mechanisms need to be seen as distinct from determinants (factors that hinder or enable a strategies impact) and strategies, activities or methods which are deployed actions. To help think about these components together,* strategies target determinants by activating related mechanisms.*^5,6^ Mechanisms can often get overlooked and are understudied^7^ as they can operate under the surface through individual beliefs and attitudes, institutionalized processes, and organizational cultures. However, these mechanisms have been noted in the implementation science literature as crucial for any type of transformation or implementation effort,^6^ including the work of innovation building and embedding.^8^

 Early in Caddedu and colleagues’ review they suggest that EDI is driven by being “frontline staff-led” and being “open and collaborative.” The authors suggest these are the two mechanisms that are needed to put EDI into practice, however, from my view, this review uncovers four important mechanisms that have the potential to drive wider efforts at large-system transformation as well.

## Mechanism #1: Empowerment

 This review found that to support EDI in healthcare organizations there needs to be a shift in power towards the employees. A shift in power like this is akin to the concept of “employee empowerment” which “involves the workforce being provided with a greater degree of flexibility and more freedom to make decisions relating to work”^2^ (p. 40). Importantly empowerment has both a structural dimension (providing support, resources and responsibility), as well as a psychological dimension (employees perceptions and beliefs regarding the power they have).^9^ Caddedu et al suggest that empowerment requires a more cooperative style of leadership, which resonates strongly with a *distributed leadership* approach which sees leadership spread amongst multiple individuals^10^; although the authors do not go all the way to suggest these more distributed power approaches. Distributive leadership fundamentally requires a shift in power to allow multiple individuals to lead from different places within an organization.^11^ The empirical literature has found that large-system transformation efforts similarly require a shift in power and autonomy. In their realist review of health system transformation, Best et al suggest the first “simple rule” of large-system transformation is that these efforts are best “ignited” through distributed leadership.^3^ Francis-Auton et al confirm that simple rule in their realist evaluation of large-system transformation, finding distributed leadership to be a critical enabler to efforts in New South Wales, Australia. In this evaluation, distributed leadership triggered a sense of ownership and control, and shared responsibility.^4^ As such, putting in processes and structures that that activate employee empowerment, as well as supporting the perception by employees that they have shared responsibility, found to drive EDI adoption at the organizational level, could potentially act as a catalyst for broader transformation efforts as well.

## Mechanisms #2 and #3: Adaptability and Learning

 Caddedu et al identify adaptation and learning as key enablers of EDI, both of which have been identified as key mechanisms in the wider implementation and innovation literatures. From these literatures adapting mechanisms can be defined as “achieving a better fit between the innovation and a given local context”^8^ (p. 239). Theoretical frameworks like the Dynamic Sustainability Framework,^12^ highlight the importance of innovation adaptation to local needs and contexts to ensure ongoing use and implementation success. Beyond the adaptation of an innovation itself, the organization needs to be able to receive and implement the innovation which can require adaptive capacity; the “underlying ability of a system, team, or organization to perform adaptations.”^13^ In their review of adaptation and innovation, Lyng et al^13^ found that long-term adaptation of an organization (like that required for large-system transformations) requires, among other things, openness to learning, yet another enabler found in Caddedu and colleagues’ review.

 Complex Adaptive Systems theory approaches to large-system transformation suggest that continuous monitoring and feedback occurring with a supportive learning environment are key mechanisms.^3^ Some more recent efforts towards adopting a Learning Health Systems approach to care delivery go a step further to purposefully embed knowledge generation and learning into their continuous process of growth and adaptation to meet local needs.^14^ Based on Caddedu and colleagues’ review a concerted effort towards embracing EDI would require an organization to embrace adaptability and learning, which are also mechanisms linked to large-system transformation efforts.

## Mechanism #4: Meaningfulness

 Caddedu and colleagues found that being “front-line staff led” and being “open and collaborative” are critical mechanisms to EDI. Bottom-up and hybrid models of EDI align with the aims of co-design and co-creation in which “end users” work with designers and system leaders to develop new models of care.^15^ Work in the areas of participatory co-design and co-creation has shown how involving end-users in the design and development of a new process or model can help individuals feel heard and valued, and helps to derive a sense of shared meaning amongst those engaging with the intervention.^16^ Deeply involving those who will ultimately be asked to change behaviours and actions when an innovation is put into practice, effectively embeds what is most meaningful and valuable to those individuals into the foundational fabric of the innovation. Bottom-up and hybrid approaches in which employees are part of the identification of the problem and solution are more closely aligned to these co-design methods, whereas top-down models, where the problem to be solved is identified by organizational leaders first, may focus on challenges that do not resonate with lived experiences of employees.

 Large-system transformations similarly require alignment around what is perceived as meaningful and valuable to all those involved in the change, potentially requiring a “shared mental model” around the purpose and benefits of a change.^4^ Furthermore, connecting new innovations to what is meaningful and valuable to those who will need to adopt the innovation can be important to ensure implementation success as meaningfulness and perceived value are mechanisms that can drive behaviour.^17^ In these ways, the hybrid approach to EDI, in which employees at the front-line work together with leadership to identify important problems to solve and devise solutions that make sense to everyone involved in the transformation may be most successful for not just EDI, but for larger system transformation.

## Why We Need EDI for People as well as Transformation

 This commentary has focused on the potential power of EDI to support large-system transformation through activation of mechanisms discovered in Caddedu and colleagues’ review. It is important to note, however that the business of large-system transformation is a complex one that requires ongoing communication and coordination processes, and interrelated drivers to help systems continue to move forward.^3,4^ Even with applying EDI approaches that can improve shared understanding and value (shown to enable large-transformation efforts^3^), it is unlikely to offer a transformation silver bullet. However, in the current climate of a widespread health human-resource crisis in which multiple global health systems are struggling to sufficiently staff their organizations,^18^ EDI may play an even more important role in supporting staff satisfaction and well-being. Caddedu et al find that EDI contributes to an increase in “vitality, satisfaction, and empowerment” amongst front-line staff. What is often heard in times of resource constraint is there simply is no time to engage in innovation activities or support staff’s ability to participate in innovation work. While this approach can be prudent, and ensure work as usual can get done, it generally assumes the time needed for innovation will only add burden. However, Caddedu and colleagues’ review shows that creating a supportive organizational environment for EDI can not only improve organizational processes but can also build a sense of meaning and resiliency for employees. This finding will hopefully encourage organizational leaders to see EDI not as taking time “away” from work, but as time that advances the organization and helps to create a culture of creativity and liveliness that not only improves outcomes, but may also spark joy.

## Ethical issues

 Not applicable.

## Conflicts of interest

 Author declares that she has no conflicts of interest.
